# Supplementary material for: Thrombotic Microangiopathy among Hospitalized Patients with Systemic Lupus Erythematosus in the United States
Source: Diseases. 2020 Dec 24;9(1):3. doi: 10.3390/diseases9010003 (PMC7838946; doi:10.3390/diseases9010003)
Supplement: Supplementary file 1 [file diseases-09-00003-s001.pdf]

Table S1 ICD-9 codes

Systemic lupus erythematosus	710.0
Thrombotic microangiopathy	446.6
Hypertension	401.1, 401.9, 642.00–642.24, 401.0, 402.00– 405.99, 437.2, 642.10–624.24, 642.70–642.94
Dyslipidemia	272.xx
Chronic kidney disease	585.1, 585.2, 585.3, 585.3, 585.4, 585.5, 585.6, 585.9
Cirrhosis	456.xx, 567.23, 571.xx,789.59
Kidney biopsy	55.23, 55.24
Therapeutic plasmapheresis	99.7, 99.71, 99.79
Invasive mechanical ventilation	96.70-96.73
Renal replacement therapy	39.95
Hemodialysis	39.95, v45.1, v56.0, v56.1
Peritoneal dialysis	54.98, v56.2, v56.32
Kidney transplant	55.6, 55.69
Hemoptysis	786.3x, 770.3
Pleural effusion/pleuritis	511.xx
Pericarditis, myocarditis, endocarditis	420.xx, 422.xx, 424.91
Glomerulonephritis	580.xx-586.xx, 791.0
Encephalitis, myelitis, encephalopathy	323.xx, 348.3x
Hemolytic anemia	283.xx, 285.xx
Thrombocytopenia	287.3x, 287.4x, 287.5
Pneumonia	480.xx-486.xx
Urinary tract infection	599.0
Sepsis	038.0, 038.10, 038.11, 038.19, 038.2, 038.3, 038.4, 038.40, 038.41, 038.42, 038.43, 038.44, 038.49, 038.8, 038.9, 790.7, 117.9, 112.5, 115.04, 115.14, 115.94, 112.81, 112.83, 003.1, 003.21, 036.2, 036.3, 036.0, 036.1, 036.42, 020.2, 022.3, 098.89, 098.84, 098.82, 995.92, 785.52
Ischemic stroke	433.xx, 434.xx, 435.xx, 436.00, 38.12, 39.51, 39.52, 39.72, 39.79, 88.41, 99.10
Seizure	345.00-345.91, 780.3-780.39, 89.14
Acute Kidney Injury	584, 584.5, 584.6, 584.7, 584.8, 584.9 (exclude 585.5, 585.6)
